# Angiogenesis: focusing on the effects of exercise in aging and cancer

**DOI:** 10.20463/jenb.2018.0020

**Published:** 2018-09-30

**Authors:** Seong-Eun Kwak, Ji-Hyun Lee, Didi Zhang, Wook Song

**Affiliations:** 1 Health and Exercise Science Laboratory, Seoul National University, Seoul Republic of Korea; 2 Institute of Sport Science, Seoul National University, Seoul Republic of Korea; 3 Institue on Aging, Seoul National University, Seoul Republic of Korea

**Keywords:** Angiogenesis, Exercise, Cancer, Aging, C2C12 cell, Aerobic exrcise

## Abstract

**[Purpose]:**

Although it is known that exercise induces angiogenesis, a clear mechanism has remained elusive due to various experimental limitations. This review presents the current status of angiogenesis-related experiments and future directions of experimentation in relation to exercise, aging, and cancer.

**[Methods]:**

We conducted a PubMed search of the available literature to identify reported exercise related changes of angiogenic factors obtained *in vitro* using C2C12 cells and endothelial cells, and *in vivo* using animal experiments and in clinical studies.

**[Results]:**

Exercise induced angiogenesis under normal conditions. Aging decreased angiogenic factors and increased during exercise. On the other hand, in cancer, the results indicate that angiogenic factors tend to increase in general, and that the effects of exercise need to be studied more. The exact mechanism remains unclear.

**[Conclusion]:**

The effect of exercise on angiogenesis appears positive. Both resistance and aerobic exercise have positive effects, but many evidences suggest that the effects are more pronounced with aerobic exercise. Further research on the precise mechanism(s) is necessary. It is expected that these studies will include models of aging and cancer.

## INTRODUCTION

The blood vessels of humans are typically in a static state. Except for the early developmental period, scarring, and during the menstrual cycle, almost no angiogenesis occurs^1^. However, many recent studies have indicated that various conditions affect angiogenesis of blood vessels.

Especially, exercise and cancer affect angiogenesis, generally by promoting angiogenesis, in contrast to aging, which decreases angiogenesis^2-4^. Angiogenesis can be viewed as a phenomenon linking exercise, aging, and cancer, and the same time being affected by these aspects^1-6^. However, although exercise, aging, and cancer can be concurrent, little research has been done on them.

Factors that regulate angiogenesis are termed angiogenic factors; they are important in inducing the formation of blood vessels in the quiescent period, and involves cytokines that include vascular endothelial growth factor (VEGF), fibroblast growth factor (FGF), and hypoxia-inducible factors (HIFs)^7,8^. Endothelial cells are directly involved in angiogenesis and act directly with angiogenic factors via tyrosine kinase receptors to regulate angiogenesis_9_. In addition, skeletal muscle cells secrete an angiogenic factor that affects angiogenesis^10^. In particular, the vascular molecules that angiogenic factors directly interact with provide nutrient support to skeletal muscles^11^. Thus, angiogenic factors are very important in skeletal muscle.

This review provides and explanation of the correlation between angiogenic factors, exercise, and aging in endothelial and skeletal muscle cells. In particular, we focus on studies that have examined the effect of exercise on endothelial cells, rather than skeletal muscle. The data may have significant meaning concerning angiogenesis in skeletal muscle. In addition, because cancer also affects angiogenesis, we examine the relationship between exercise and cancer in terms of angiogenesis.

**Fig. 1.**
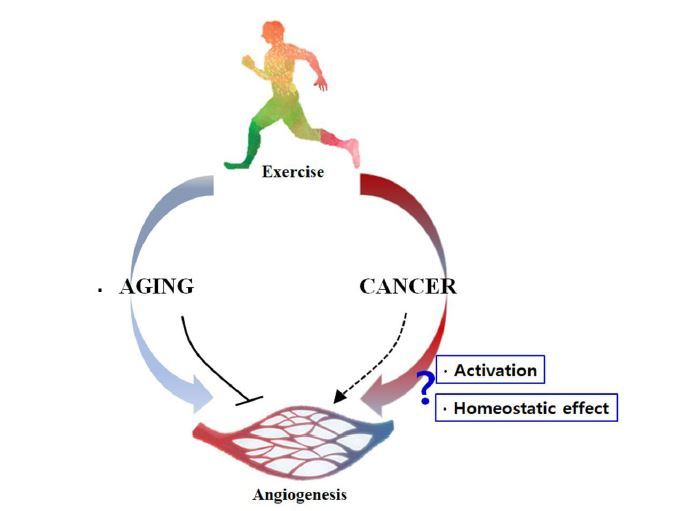
Summary of the study. --> denotes activation and denotes inhibition.

### Exercise and Angiogenesis

Exercise has a positive effect on long-term cardiovascular disease ^12^. In particular, exercise plays a major role in alleviating vascular resistance and stiffness^13^.

A number of in vitro, cell-based studies have been conducted to evaluate the changes in the pattern of angiogenesis pattern after exercise using endothelial cells to try to mimic the in vivo condition^14,15^. Endothelial cells are the major cells forming blood vessels^16^, and are influenced by angiogenic factors that bind to tyrosine kinase receptors^9^. The identification of angiogenesis-related molecules that mimic exercise in endothelial cells is directly related to endothelial vascular formation. An example is the influence of 5-aminoimidazole-4-carboxamide ribonucleotide (AICAR) on human umbilical vein endothelial cells (HUVEC) and rat myocardial microvascular endothelial cells (MMEC)^14,15,17^. In the case of HUVEC cells, AICAR treatment decreased the amount of fatty acids and lactate^14^, In case of MMEC, AICAR treatment increased AMPK and VEGF mRNA expression^17^. Bovine capillary endothelial (BCE) cells are also representative endothelial cells^18^. BCE cells were treated with soleus muscle extracts of rats treated with 15 weeks of exercise. Cell fluorescence was increased compared to the cells not treated with extracts. These results indicated that endothelial cell growth can be induced by exercise. The significance of this result is that exercise promotes angiogenesis by promoting the proliferation of endothelial cells that directly form blood vessels^18^.

C2C12 cells are representative skeletal muscle cells^19^. There are many ways to mimic the mechanism of exercise in C2C12 cells. The most commonly used methods include AICAR-based chemical methods. Other methods include physical stimulation of skeletal muscle cells through electrical stimulation^20,21^. As a result of treatment with AICAR, phosphorylation of AMPK and its subordinate signals were induced. Thus, the expression level of VEGF was confirmed by real-time PCR, The amount of VEGF expression was increased in proportion to the amount of AICAR. This result was almost identical to that obtained by insulin treatment. Treatment with AICAR pathway inhibitor and insulin pathway inhibitor confirmed that the mechanism of VEGF promoted by exercise and by insulin were different from those of AICAR and insulin^22^. VEGF expression using AICAR to mimic exercise resulted from the increased mRNA stability through AMPK, and VEGF expression by insulin was the result of increased transcription levels through phosphoinositol-3-kinase^22^. When electric pulse stimulation was applied to C2C12 cells, signals including extracellular signal-regulated kinase 1/2 and 5, c-Jun N-terminal kinase, and JNK and ATF, which are influenced by exercise, were activated as was glucose transporter type 4^23^. When primary muscle cells were treated with EPS , the content of VEGF, which affects angiogenesis, was significantly increased. The increase was not immediate but occurred over the ensuing 24 hours. Fibroblast growth factor (FGF) increased only after EPS treatment compared to control group^24^. The observations suggest that the secretion of angiogenic factor in the muscles due to exercise continues both immediately after exercise and for some time after exercise (Table 1).

**Table 1.** Exercise mimetic effects on cellular models.

Cultured Cell Type	Exercise mimic	Molecular change
HUVEC^14^ MMEC^17^	AICAR treatment	Fatty acids↓ Lactate↓ VEGF mRNA↑
BCE cells^18^	Exercise treated mouse’s soleus muscle extract ^18^	DNA fluorescence↑
C2C12 cells	0.5 mM AICAR^22^	VEGF mRNA 1.5-fold↑
	2 mM AICAR^22^	VEGF mRNA 2-fold↑
	EPS treatment^23^	Erk 5↑ Erk 1/2↑ JNK↑ ATF↑ GLUT4↑
Primary muscle cell^24^	EPS treatment	VEGF↑ FGF↑

HUVEC; Human umbilical vein endothelial, MMEC; Rat myocardial microvascular endothelial cell, BCE; Bovine capillary endothelial, AICAR; Aminoimidazole-4-carboxamide ribonucleotide

In an in vivo experiment, obese and normal weight male Zucker rats were exercised at increase exercise intensity five times a week for 8 weeks^25^. The level of VEGF-A was increased in the epididymal adipose tissues of all rats^25^. In this case, angiogenesis in the adipose tissue plays a role in inhibiting hypoxia and inflammation, mitigating cardiovascular disease^26^. In the case of 4-week-old Sprague-Dawley rats, mRNA expression levels of angiogenic factors that included VEGF, mammalian target of rapamycin (mTOR), and mTOR-C in the skeletal muscle increased after treadmill exercise^27^. To examine the effects of the resistance exercise, a clinical study was performed in which serum was collected and analyzed for 8 weeks after an exercise regimen. The angiogenic factors VEGF and angiopoietin 1 (Ang1) were significantly increased^28^. The in vivo and clinical data confirmed the inducing effect of angiogenesis by exercise and the appropriate exercise intensity for inducing angiogenesis (Table 2).

**Table 2.** Exercise effects in *in vivo* models

Model	Exercise	Molecular change
Male Zucker rat; Obesity, normal Model^25^	20 m/min for 60 min, 5 days each week for 8 weeks	In adipose tissue, VEGF-A level↑
Sprague-Dawley rats^27^	5 days each week for 8 weeks, incremental treadmill exercise	In skeletal muscle, VEGF↑ mTOR-C↑ mTOR↑ (mRNA expression)

### Aging and Angiogenesis

Aging is associated with diminished genetic processes, mitochondrial degeneration, cell death, and loss of enzyme activities ^29^. In particular, in some tissues associated with angiogenesis, VEGF, which is the most basic and dominant regulator, decreases with aging^30^. VEGF usually acts on endothelial nitric oxide synthase (eNOS) to induce vasodilation and angiogenesis. During senescence, eNOS activity decreases due to oxidative stress, decreased activity of sirtuin 1 (SIRT1), and decreased estrogen production^6^. In blood vessel, capillary density and formation, which are important in the removal of metabolic byproducts and oxygen transport, are directly affected by angiogenic factors^31^. We examined the angiogenesis patterns in human dermal microvascular endothelial cells (HMVECs) using aged and neonatal HMVECs, and found that capillary formation in aged HMVECs was reduced compared to neonatal cells, and that VEGF mRNA and protein levels were significantly decreased in aged HMVECs^30^.

Doxorubicin is used for chemotherapy in cancer patients. When administered to C2C12 cells, doxorubicin increases protein degradation through the production of reactive oxygen species (ROS) of mitochondria and induces catabolism^32^. Elevated ROS levels and increased protein degradation due to mitochondrial dysfunction in muscle may be a hallmark of aging^33^. Another study described that doxorubicin induces the production of urokinase receptor (uPAR), which regulates telomeric repeat binding factor 2 (TRF2), in turn causing ubiquitination and proteasomal degradation^34^. Therefore, by treating C2C12 cells with doxorubicin, it is possible to mimic the environment of aged C2C12 cells. Until now, there has been no comparative study of changes in VEGF, Ang1, platelet-derived growth factor (PDGF), and basic FGF (bFGF), but the overall effect of aging is expected to reduce angiogenic factors.

In vivo studies have shown that capillary density, which can be calculated as the capillary area per fiber area, decreases with age^35^. Comparison of the capillary density of 5, 13, and 25 month old rats with plantaris muscle has revealed the decreased capillary area with aging^36^. Another study compared hind limb capillary densities of 12-week-old mice and 24-month-old mice to investigate the influence of aging. The capillary density of the 24-month-old mice was twice that of the younger mice^37^.

In a clinical 12-year longitudinal study of males, the pre-capillary density was higher than post-capillary density, and the ratio was reduced from 1.39 to 1.08^38^. Comparing the capillary density of type 2A muscles through the vastus lateralis in subjects 19-25 and 62-72-years-of-age group confirmed a significant decrease in capillary density with age^39^. Another study compared the expression levels of the angiogenic inducers bFGF and PDGF in the venous wall of young and elderly patients. Both molecules were significantly reduced in the elderly^40^. Still another study compared the ratio of VEGF/β-actin by analyzing the gastrocnemius muscle of 22-28-year-old adults and elderly women aged 60-85 years. The ratio value was significantly reduced in the elderly women^41^.

### Effect of Exercise on Angiogenesis During Aging

As mentioned earlier, aging is associated with reduced angiogenesis in muscle tissue and endothelial cells, with consequent reduction of angiogenic factors^35^. On the other hand, since exercise induces angiogenesis, it can be assumed that the exercise will lead to an improvement in angiogenesis (which could be examined in a model of aging). In relation to this, the ratio of capillary density in variable muscle type and ages was significantly increased after exercise, even when compared with the effect of exercise in younger and elderly subjects, which revealed no significant difference attributable to age^42^. In addition, the levels of expression of VEGF protein and RNA in human vastus lateralis biopsies were significantly increased in the elderly during exercise^42^. The level of VEGF was significantly elevated upon 50 minutes of mild intensity exercise in another group of elderly subjects^39^. In another study, eight women aged 57-76 years or 20-29 years underwent low intensity exercise four times a week for one hour. Both groups displayed similarly increased levels of vastus lateralis VEGF protein^43^. The collective observations indicate the age-related reduction in angiogenesis can be overcome by exercise, regardless of gender [Table 3].

**Table 3.** Exercise effects in clinical studies

Subjects	Exercise	Molecular change
Young (mean age 25 years) Old (mean age 60 years) ^42^	4 days per week, with VO2 max 65 % for 8 weeks	In vastus laterals, capillary density ↑ VEGF RNA expression↑
Old group ^39^	50 min , VO_2_ max 50 % of cycle ergometer	VEGF↑
Aged female (57-76 years) Young female (20-29 years) ^43^	8 week, 65% Vo_2_ max, 1 hour 4 days each week	Old group VEGF unchanged Young group VEGF ↑

### Effects of Cancer and Exercise on Angiogenesis

As with exercise and aging, cancer also affects the body’s metabolism. In the case of cancer, angiogenesis was thought to be promoted after the formation of tumors. It is now believed that the reverse is the case—the tumor promoted by angiogenesis contains enlarged blood vessels, which can activate the Warburg effect^1,44^. Therefore, unlike the previous view, in cancer, angiogenesis plays a role in accelerating tumor progression. Despite the importance of angiogenesis, there has been little research on the relationship between cancer and exercise in terms of angiogenesis. While the effects can vary according to the type of cancer, exercise is effective in alleviating cancer^45,46^. A consideration of the mechanisms of cancer and exercise in terms of angiogenic factors indicates that both may induce angiogenesis, which contradicts the view that the effects of cancer are lessened by exercise^47,48^. These contradictory views have not been well studied. However, since exercise has a major role in maintaining homeostasis, further studies will surely be done to explore the influence of exercise on angiogenesis in cancer patients and in cancer cells.

### Limitations and Further study

Angiogenesis and related phenomena have been well studied in some aspects and incompletely studied in other aspects. The latter especially includes research related to aging and cancer.

It is difficult to study the mechanism of aging due to compatibility issues with the model used. In the end, the purpose of aging model research is for clinical study. Mus musculus is a stalwart aging model. However, mice are grown in extremely limited environment, which limits the generalization of the results to aging humans^49^. In addition, in vivo studies are limited concerning their capacity to establish mechanisms of exercise effects on aging-induced models, so cell-based studies are needed. However, in cell-based studies, aging is defined as an accumulation of damage or errors in the cellular level and an insufficiency of self-repair^50^. Definitions of aging and aging model building at a cellular level are needed for studies of mechanism.

### Summary

Angiogenesis has many effects on the human body. In particular, it responds to external stimuli and helps maintain homeostasis. Exercise induces angiogenesis, which in turn changes the patterns of the molecules involved. In cell-based studies, in vivo models, and clinical studies, angiogenesis is inhibited by aging and senescence, and the expressions of factors induced by angiogenesis, such as VEGF, are decreased. Although it has been shown that angiogenic factors are reduced in aging models or the elderly with exercise, there mechanism has not been elucidated in cell-based studies and in vivo models. This is because a suitable aging model has not been properly established and because the reliability of the results of studies to date can be questioned based on the characteristics of aging. In the case of cancer-related studies, most of the tissues have a tendency to display accelerated angiogenesis when tumors arise. Therefore, further studies are needed to determine how exercise affects angiogenesis in cancer. These studies are challenging because it is not easy to perform exercise with cancer patients and because of the challenges in establishing cell models or in vivo models. The establishment of proper models is necessary.
